# Designer nanoparticle: nanobiotechnology tool for cell biology

**DOI:** 10.1186/s40580-016-0082-x

**Published:** 2016-09-15

**Authors:** Deepak B. Thimiri Govinda Raj, Niamat Ali Khan

**Affiliations:** 1 European Molecular Biology Laboratory (EMBL), Grenoble Outstation and Unit of Virus Host-Cell Interactions (UVHCI), UJF-EMBL-CNRS, UMR 5233 Grenoble, France; 2 Envirotransgene Bio-solutions Global, Chennai, India; 3 Biotechnology Centre for Oslo, Centre for Molecular Medicine Norway (NCMM), P.O. Box 1137, Blindern, 0318 Oslo, Norway; 4 Laboratory of Lipid Metabolism and Cancer, O&N I, Herestraat 49, Box 902, 3000 Louvain, Belgium

**Keywords:** Nanoparticles, Subcellular proteomics, Nanobiotechnology, Systems biology

## Abstract

This article discusses the use of nanotechnology for subcellular compartment isolation and its application towards subcellular omics. This technology review significantly contributes to our understanding on use of nanotechnology for subcellular systems biology. Here we elaborate nanobiotechnology approach of using superparamagnetic nanoparticles (SPMNPs) optimized with different surface coatings for subcellular organelle isolation. Using pulse-chase approach, we review that SPMNPs interacted differently with the cell depending on its surface functionalization. The article focuses on the use of functionalized-SPMNPs as a nanobiotechnology tool to isolate high quality (both purity and yield) plasma membranes and endosomes or lysosomes. Such nanobiotechnology tool can be applied in generating subcellular compartment inventories. As a future perspective, this strategy could be applied in areas such as immunology, cancer and stem cell research.

## Background

Nanotechnology is defined as molecular engineering of functional systems for generating high performance technologies for both research and industry [1, 2]. Specifically, use of molecular engineering of biological systems resulted in emergence of Nanobiotechnology [3]. Some of the nanobiotechnology applications include (a) nanoscopy for bio imaging [4–7]; (b) nanoparticle for subcellular fractionation [8]; (c) nanoparticle for drug delivery, vaccine [9]; (d) nanoparticle for cancer therapy by hyperthermia [10]; (e) nanomaterials for tissue engineering and artificial/synthetic organ generation [11]; (f) nanotechnology for cell tracking [12, 13]; and (g) nanotechnology for large-scale data generation [14]. Particularly, use of nanotechnology in generating subcellular omics is less studied and understood. This article focuses on nanobiotechnology strategy for organelle isolation and also deciphers innovative approaches for omics analysis. Using physical properties, its nanoparticle-cell interaction and endocytosis machinery, we propose the nanobiotechnology strategy that has robust advantages in isolating subcellular compartments [15]. Due to such advantages, it is possible to isolate subcellular compartments in native and physiological conditions. In this article, we also case study the impact of nano-biotechnology tool for subcellular omics analysis.

## Subcellular omics

To generate subcellular omics datasets, it is essential to understand the locality and functional activity of proteins in given eukaryotic cell [16]. It is well-known that proteins are spatially distributed and localized function [17]. It has been reported that majority of the mature glycosylated protein (for example: Nicastrin) are present in post-Golgi compartments like plasma membrane, endosomes or lysosome and immature glycosylated protein are present in the pre-Golgi compartments [18]. Similarly, cholesterol is predominately present in the cell membrane at the level of 90 % of the total cholesterol level in cell extract [19]. While performing proteomics or lipidomics in total cell extract or single cell omics, there is a high possibility of reduced spatial and localized distribution of proteins and lipids in any given cell. This has led to a major interest for subcellular omics such as plasma membrane and endosomal compartments like endosomes and lysosomes [20]. This is due to several scientific findings which confirm that the majority of protein functional activity and substrate cleavage occurs at cell surface and endosomal compartments (Table 1) [21]. Similarly by isolating plasma membrane or endosomes, it is possible to generate proteomics, glycomics and lipidomics for these organelles [22]. By compiling obtained omics (proteomics, glycomics, and lipidomics), for different organelles, comprehensive whole cell omics can be generated both under native and altered conditions [23–26]. The key factor for generating comprehensive omics datasets s is to isolate subcellular compartments with high purity and yield. Several types of fractionation methodologies have been applied for organelle isolation for subcellular omics analysis. In the technology review, we elaborate advantages among different subcellular compartmental isolation and how nanobiotechnology strategy is superior in isolating plasma membrane and endosomes as in Tables 2 and 3.

**Table 1:** List of subcellular organelles and their functions

Subcellular Organelles	Isolation techniques
Cell wall	Gradient centrifugation
Chloroplast	Gradient centrifugation antibody based pull-down assay, SPMNPS: Tag-anti-tag; Antibody conjugated; Biotin-streptavidin
Cilia and flagella	Gradient centrifugation Antibody based pull-down assay; Tag-anti-tag; Antibody conjugated; Biotin-streptavidin
Cytoplasm	Gradient centrifugation antibody based pull-down assay; Tag-anti-tagged; Antibody conjugated; Biotin-streptavidin tagged SPMNPs
Cytoskeleton	Gradient centrifugation antibody based pull-down assay; Tag-anti-tagged; Antibody conjugated; Biotin-streptavidin SPMNPs
Early endosomes	Gradient centrifugation SPMNP isolation assay antibody based pull-down assay; Biotin-streptavidin tagged SPMNPs; antibody-SPMNPs; Negatively charged lipid-SPMNPs
Endoplasmic reticulum (ER)—rough or smooth	Gradient centrifugation antibody based pull-down assay; Tag-anti-tag; Antibody conjugated; Biotin-streptavidin tagged SPMNPs
Golgi apparatus	Gradient centrifugation antibody based pull-down assay; Tag-anti-tagged; Antibody conjugated; Biotin-streptavidin tagged SPMNPs
Late endosomes	Gradient centrifugation SPMNP isolation assay; Biotin-streptavidin; antibody-SPMNPs; Negatively charged SPMNPs
Multi-vesicular bodies	Gradient centrifugation SPMNP isolation assay; antibody based pull-down assay; Biotin-streptavidin; antibody-SPMNPs
Nucleus	Gradient centrifugation antibody based pull-down assay; Tag-anti-tagged; Antibody conjugated; Biotin-streptavidin
Peroxisomes	Gradient centrifugation antibody based pull-down assay; Tag-anti-tagged; Antibody conjugated; Biotin-streptavidin tagged SPMNPs
Phagosomes	Gradient centrifugation SPMNP isolation assay Antibody based pull-down assay; Biotin-streptavidin; antibody SPMNPs
Lysosomes	Gradient centrifugation SPMNP isolation assay Antibody based pull-down assay; Biotin-streptavidin tagged SPMNPs; antibody
Plasma Membrane or Cell Membrane	Gradient centrifugation; Cationic silica beads; SPMNP isolation; antibody conjugated; Biotin-Streptavidin; lectin–SPMNPs
Ribosomes	Gradient centrifugation; pull -down assay; anti-S10/anti–EF-Tu-SPMNPs; Biotin-streptavidin; antibody- SPMNPs
Lipid rafts	Gradient centrifugation antibody based pull-down assay; Tag-antitagged-SPMNPs; Biotin-streptavidin; protein conjugated magnetic isolation
Secretory granules or vesicles	Gradient centrifugation antibody based pull-down assay; Tag-antitagged SPMNPs; Biotin-streptavidin-SPMNPs
Synaptosomes	Gradient centrifugation antibody based pull-down assay; Tag-anti-tag SPMNPs; Antibody conjugated; Biotin-streptavidin SPMNPs
Vacuoles	Gradient centrifugation; antibody based pull-down assay; antibody conjugated magnetic nanoparticles
Mitochondria	Gradient centrifugation; pull-down assay; antibody conjugated SPMNPs; Tag-anti-tagged; Antibody conjugated; Biotin-streptavidin; Anti-TOM22 antibody tagged SPMNPs

**Table 2:** Comparison of existing technologies for plasma membrane isolation

Isolation methodologies	Advantages	Disadvantages
Density Gradient Centrifugation	Conventional method that can be used to isolate plasma membrane along with other subcellular compartments Effective method to isolate lysosomes from tissue or in vivo cell fractions Simple procedure that can be performed using an ultracentrifuge	Low yield and low purity Cannot isolate intact membrane layers Not efficient for isolating cell membrane lipids and for performing functional studies
Cationic silica based isolation	Classical method used to isolate cell membrane layers with high purity Generic method used to isolate cell membrane from tissue, in vitro and in vivo Formation of matrix by the use of polylysine crosslinker helps in the isolation of membrane layers without any breakage	Low yield Isolates only available membrane layer i:e only 50 % of cell surface of adherent cells grown on a petri dish Not robust to perform lipidomics, glycomics and native condition experiments
Cell Surface biotinylation based pull-down assay	Generic method that targets cell surface lysine residue Can be used in combination with magnetic or non-magnetic beads Can be used in combination to isolate endosomal compartments	Can isolate only available membrane layer i:e only 50 % of cell surface of adherent cells growing on a petri dish Technology has not been established to isolate cell membrane from cell suspension Requires detergent in fractionations
Antibody conjugated magnetic nanoparticle based pulldown assay	Can be used to pull down proteins after post-fractionation Can also be used to target selective micro-domains Can be used in combination with biotin-streptavidin assay	Isolates only available membrane layer (only 50 % surface of adherent cells on a petri dish Technology has not been established to isolate cell membrane from cell suspension requires detergent in fractionations
SPMNPs based plasma membrane isolation;	A novel strategy that is generic for any kind of cell systems Method does not involve use of detergent or antibody that affects the nativity of membranes Method can also use targeted plasma membrane micro-domain by using ligand-tagging Can be used to isolate protein under native conditions which can hence be used for functional studies Can isolate cell membrane lipids Can perform, first of its kind, cell membrane glycosylation High yield and high purity Can be used in combination with endosomal isolations	Technology has not be established for tissue cell membrane isolation and in vivo experiments Isolates only available membrane layer (only 50 % surface of adherent cells on a petri dish Technology has not been established to isolate cell membrane from cell suspension. Possibility exists to use this technology for cells in suspension culture

**Table 3:** Comparison of existing technologies for endosomes and lysosome isolation

Isolation methodologies	Advantages	Disadvantages
Density gradient centrifugation;	Conventional method that can be used to isolate endosomes and lysosomes along other subcellular compartments Effective method to isolate lysosomes from tissue or in vivo cell fractions Simple procedure that can be performed using an ultracentrifuge	Low yield Difficulties in separating endosomes from lysosome vesicles Difficulties in separating different endosomal and its associated vesicles
Antibody based pull-down assay	Can be used to pull down proteins after post-fractionation Can also be used to target selective endocytic uptakes Can be used in combination with biotin-streptavidin assay	Limited applicability for certain endocytosis uptake Limited yield and low purity Cannot isolate vesicles under native conditions
SPMNPs based isolation;	A novel strategy that is generic for any kind of cell systems with reasonable purity and yield Method does not involve the use of detergent or antibody that affects native conditions Method can also use targeted endosomal uptake pathway by using ligand tagged nanoparticle Can be used to isolate protein under native conditions and all endosomal uptake	Technology has not be established for isolation of vesicles form tissue cells and in vivo experiments Technology has not been established to isolate vesicles from cell suspension. Possibility exists to use this technology for cells in suspension culture

## Technology review

### Organelle fractionation and subcellular compartmental isolation

**Fig. 1:**
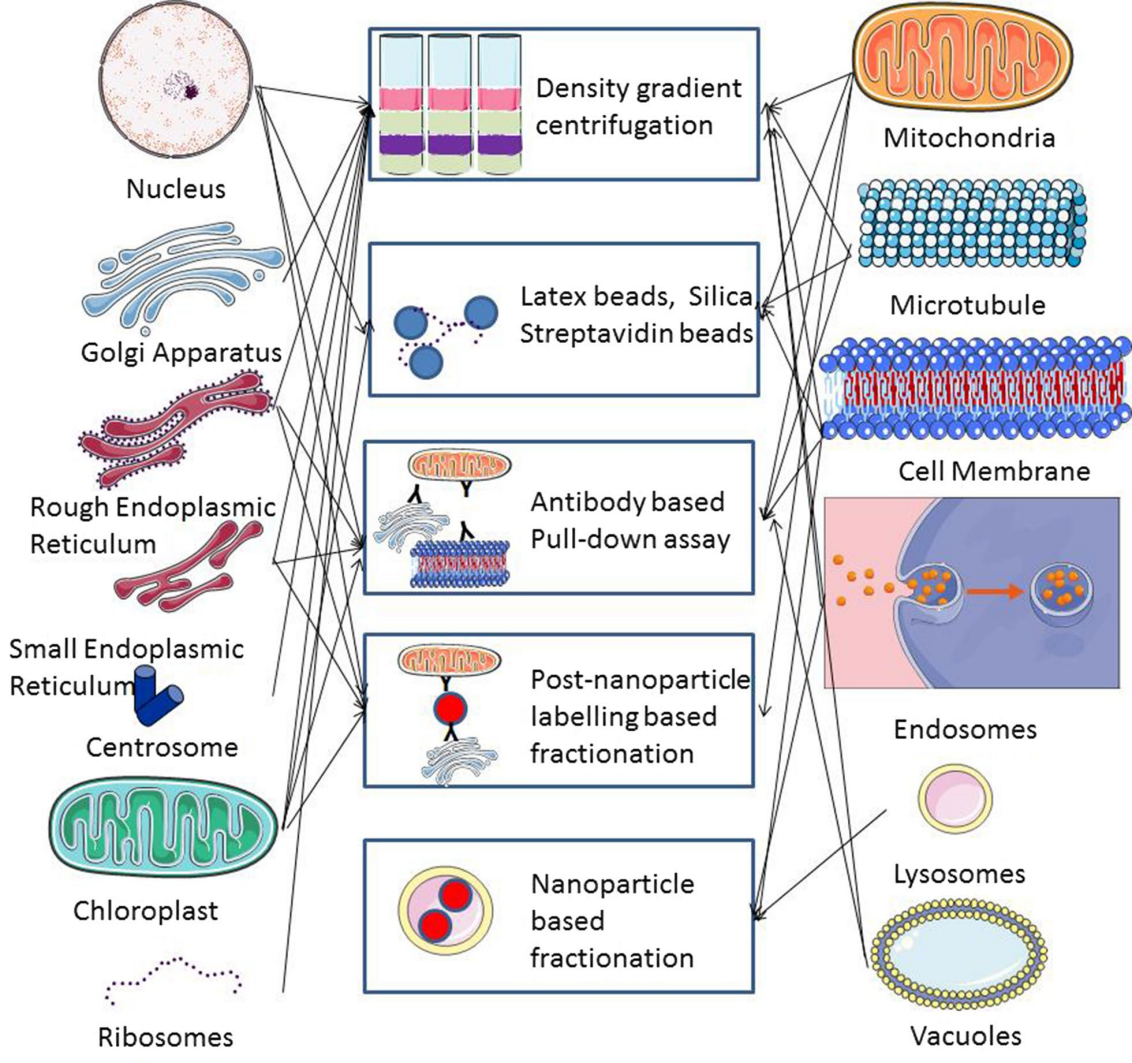
Subcellular compartments and specific purification methodology

The governing factor for organelle fractionation is high yield and high purity (Fig. 1). Most commonly used methodology is density-gradient centrifugation (sucrose-based fractionation). This method is based on principle of differential (density) equilibrium or non-equilibrium based centrifugation for organelle separation [27, 28]. Other commonly used fractionation is antibody based pulldown assay. This assay makes use of magnetic beads that are tagged with antibodies selectively targeting the subcellular compartments [29]. An example is the use of TOM22 antibodies conjugated with magnetic beads for mitochondria isolation [30, 31]. This principle is also used for post-nanoparticle labeling based fractionation. Here, organelle-specific antibody conjugated nanoparticles are used to target fractionated subcellular compartment. This technique is largely used in isolating compartments that are larger in size and less dynamic or more static such as ER, Golgi, nucleus, mitochondria and lysosomes [32]. However, the applicability of this technique is limited as it cannot be used to isolate intact cell membrane. Many methods have used charge based affinity to isolate eukaryotic cell membrane [33]. The cell membrane is negatively charged due to presence of anionic components such as proteins, lipids and carbohydrates. Hence, conventional method such as cationic latex or silica beads is used for isolating cell membrane. By using poly-lysine for crosslinking silica beads, cell membranes are isolated as cross-linked membrane layers which are disadvantage for performing functional studies [34]. An alternative method that has been used is biotin-streptavidin affinity fractionation. The main principle behind biotin-streptavidin affinity assay is based on selective binding of available lysine residues on cell surface protein by biotin molecule which is further captured by streptavidin [35]. These streptavidin tagged micro beads or magnetic beads are used to pull down cell membrane protein or protein complexes from cell fractionation. By using pulse-chase method (elaborated in the later part of the paper), biotin-streptavidin affinity can be used to isolate early, intermediate and late endosomal compartments [36]. Recently, nanoparticle based fractionation has emerged as the strategy to isolate dynamic subcellular compartments like cell membrane, endosomes and lysosome by using endocytosis machinery.

**Fig. 2:**
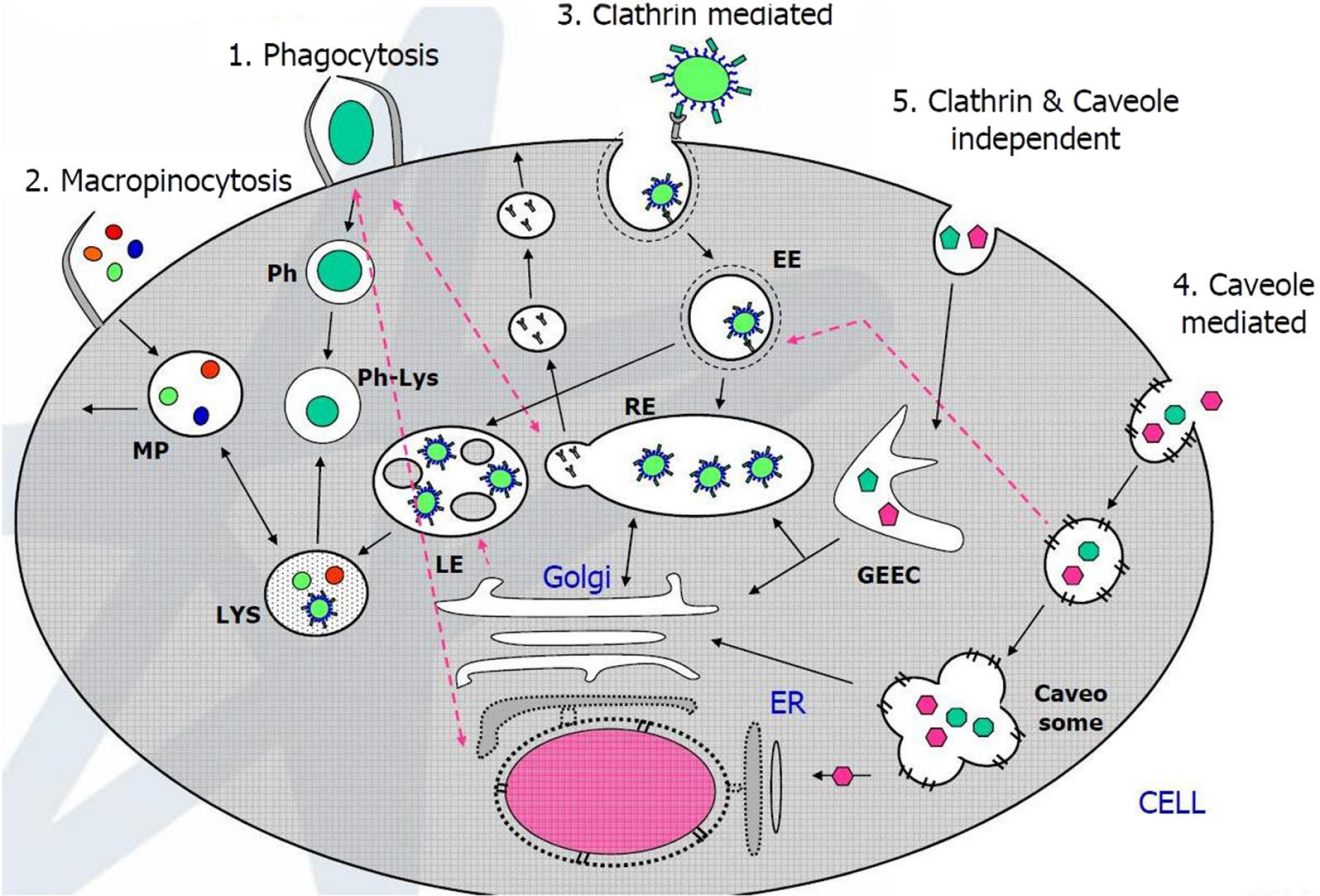
Endocytosis pathway

There are different categories of endocytosis (Fig. 2). One of the main subsets is phagocytosis that mainly involves cellular uptake of beads of size 1–100 microns using phagosome [37, 38]. Another strategy is use of pinocytosis which is further classified into (1) micropinocytosis (<1 micron) [39]; (2) clathrin-mediated pinocytosis: size ~120 nm by clathrin mediated receptor-ligand induced cellular uptake [40]; (3) caveolin-mediated pinocytosis: is cellular uptake that uses caveolin and lipid rafts in endosomal assembly with size of 20–60 nm [41]; and (4) caveolin and clathrin independent endocytosis (<90 nm) that is independent of clathrin and caveolin but includes ARF6 [42]. In macropinocytosis, there is non-specific cellular uptake, which is mainly utilized by several nanoparticles–cell interactions [43]. In clathrin mediated endocytosis, ligand coupled nanoparticle endocytosis through receptor mediated uptake mechanism [44, 45]. While for caveolin mediated endocytosis, targeting caveolin1/2 or lipid raft associated protein flotilin-1 with the specific antibody tagged nanoparticle is the commonly used approach [46]. However, when clathrin mediated endocytosis was blocked using sucrose, nanoparticle uptake was not limited, and thereby showing that there is size dependent cellular uptake of nanoparticle via caveolin mediated endocytosis [47]. Nevertheless it is clear that surface coating and size of the nanoparticle are governing factors for selective endocytosis and cellular uptake.

## Nanoparticle synthesis

**Fig. 3:**
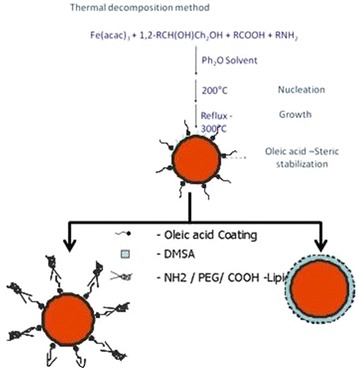
Manufacturing of water-soluble superparamagnetic nanoparticles

The surface functionalization, size, physical properties and endocytosis machinery of nanoparticle are key factors for nanobiotechnology strategy. The physical properties are dependent on the type of core–shell material [48, 49]. It is possible to govern the magnetic properties of the nanoparticle by using iron oxide or cobalt-iron oxide as a core. Here superparamagnetic properties can be achieved when nanomaterial is of size <30 nm [50]. Shell material (surface coating) which acts as an interface between core and biological environment governs the use of nanoparticle for different biological applications. Nanoparticles that are synthesized in organic phase tend to be water insoluble. They require an additional step of exchange with water soluble ligands for biological applications. Commonly used methods for synthesizing nanoparticle are (a) chemical precipitation method and (b) thermal decomposition method [51]. Thermal decomposition method is preferred for its high monodispersity (in terms of its size) and high quality yield. However, it requires additional step for water-soluble ligand exchange or addition [52, 53] (Fig. 3). Further, functionalized nanoparticle can be used for ligand coupling and bioconjugation using the free end-groups like NH_2_, COOH and -SH.

## Bioconjugation strategy for subcellular interaction

**Fig. 4:**
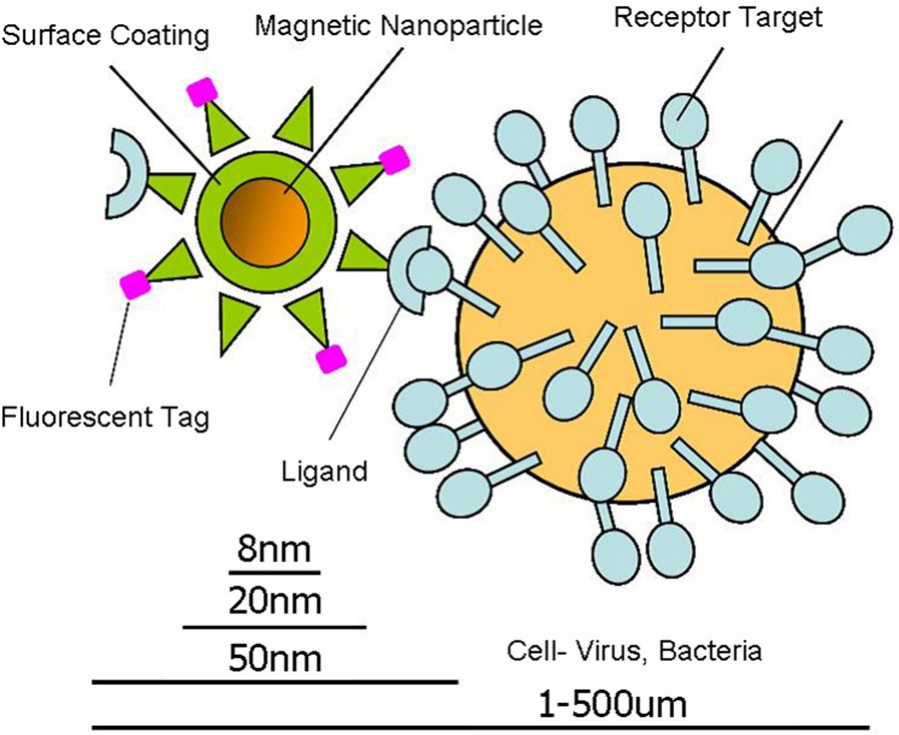
Nanoparticle-cell interaction

**Fig. 5:**
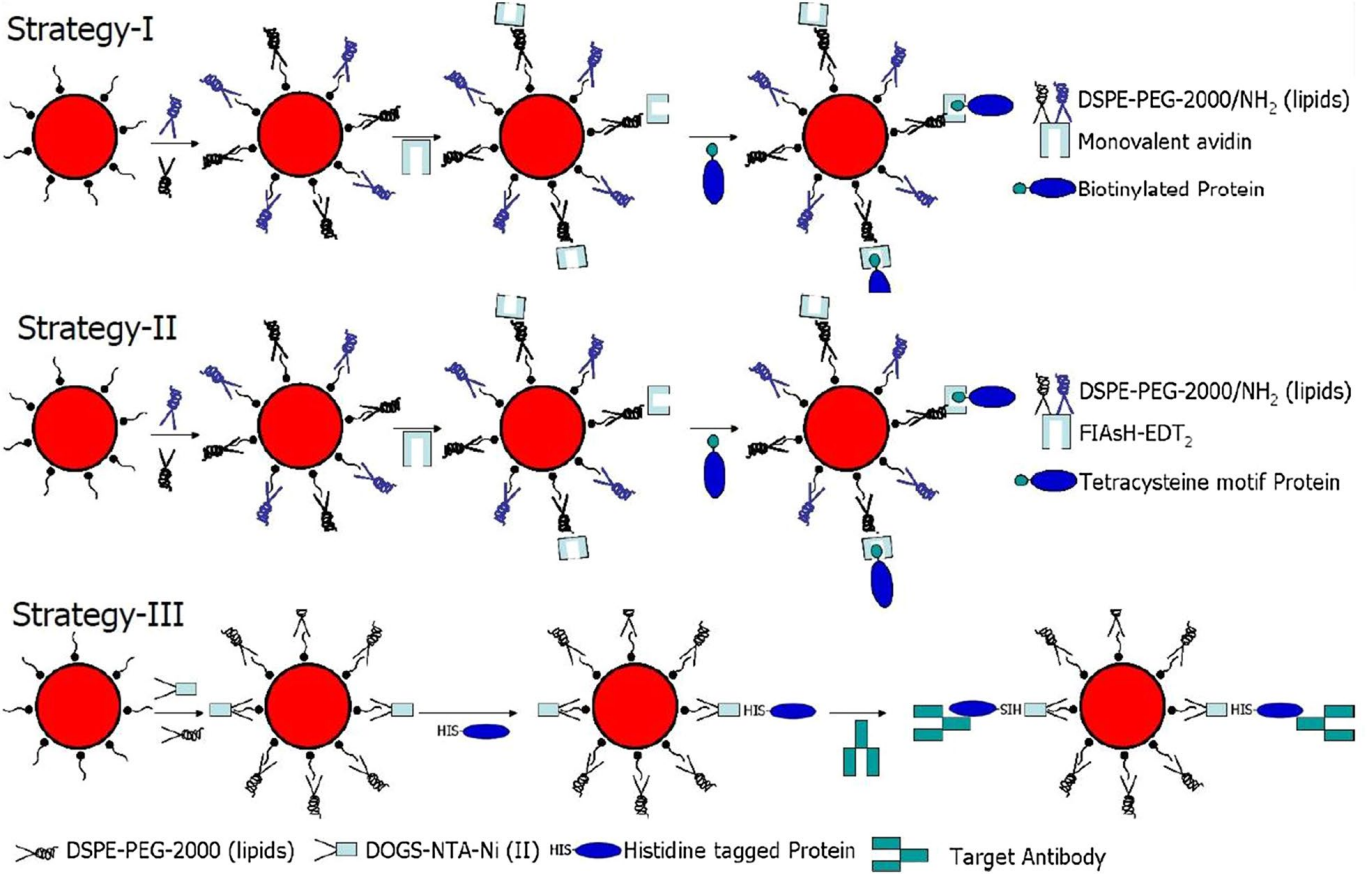
Bioconjugation strategy for nanoparticle

Surface functionalization of nanoparticle determines the kinetics behind nanoparticle-cellular uptake. Depending on the surface coating, nanoparticle can be ± charged which is determined by the presence of NH_2_ and COOH respectively. Addition of PEG group in supports biocompatibility [54] and the nanoparticle can be further functionalized by coupling with the endgroup (Fig. 4). By tagging fluorescent ligand, it is possible to perform live cell imaging and nanoparticle tracking for studying the receptor-ligand and nanoparticle-cell interaction. Depending on the target, an appropriate ligand can be selected and conjugated to the nanoparticle [55]. Ligand size and shape determines the surface area to volume ratio which is the governing factor for nanoparticle functionalization [56]. Another key factor is ligand selection. Ligand selection depends—(a) receptor that can be well internalized (for cellular trafficking); b) 1:1 ratio of ligand: nanoparticle to avoid nanoparticle crosslinking. Here we illustrate three strategies for bioconjugation of SPMNPs that are functionalized with lipids, DMSA, TMAOH (Fig. 5). First approach is to use monovalent avidin and target biotinylated protein. Second approach proposes the conjugation of FIAsH-EDT_2_ with SPMNPs. FIAsH-EDT_2_ coupled SPMNPs are used to couple tetra-cysteine containing motif proteins [57]. Third approach is to conjugate SPMNPs with DOGS-NTA-Ni (II) in order to anchor histidine-tagged protein. However, major limitation for bioconjugation is nanoparticle aggregation due to coupling reagent like glutaraldehyde. Although glutaraldehyde works very well for protein conjugation and crosslinking, there are tendency for reagent to result in multiple layer crosslinking among nanoparticles due to non-specific interaction and competitive affinity. Such multilayer crosslinking results in increase of size and change in physical properties, thereby affecting organelle isolation. Hence some key guidelines to be considered during bioconjugation of nanoparticles are (1) retaining size and stability of nanoparticle; (2) performing sequential bioconjugation; (3) implementing biocompatible surface functionalization; and (4) finally, a strategy that monitors protein association/disassociation with the nanoparticle.

## Pulse-chase methodology

**Fig. 6:**
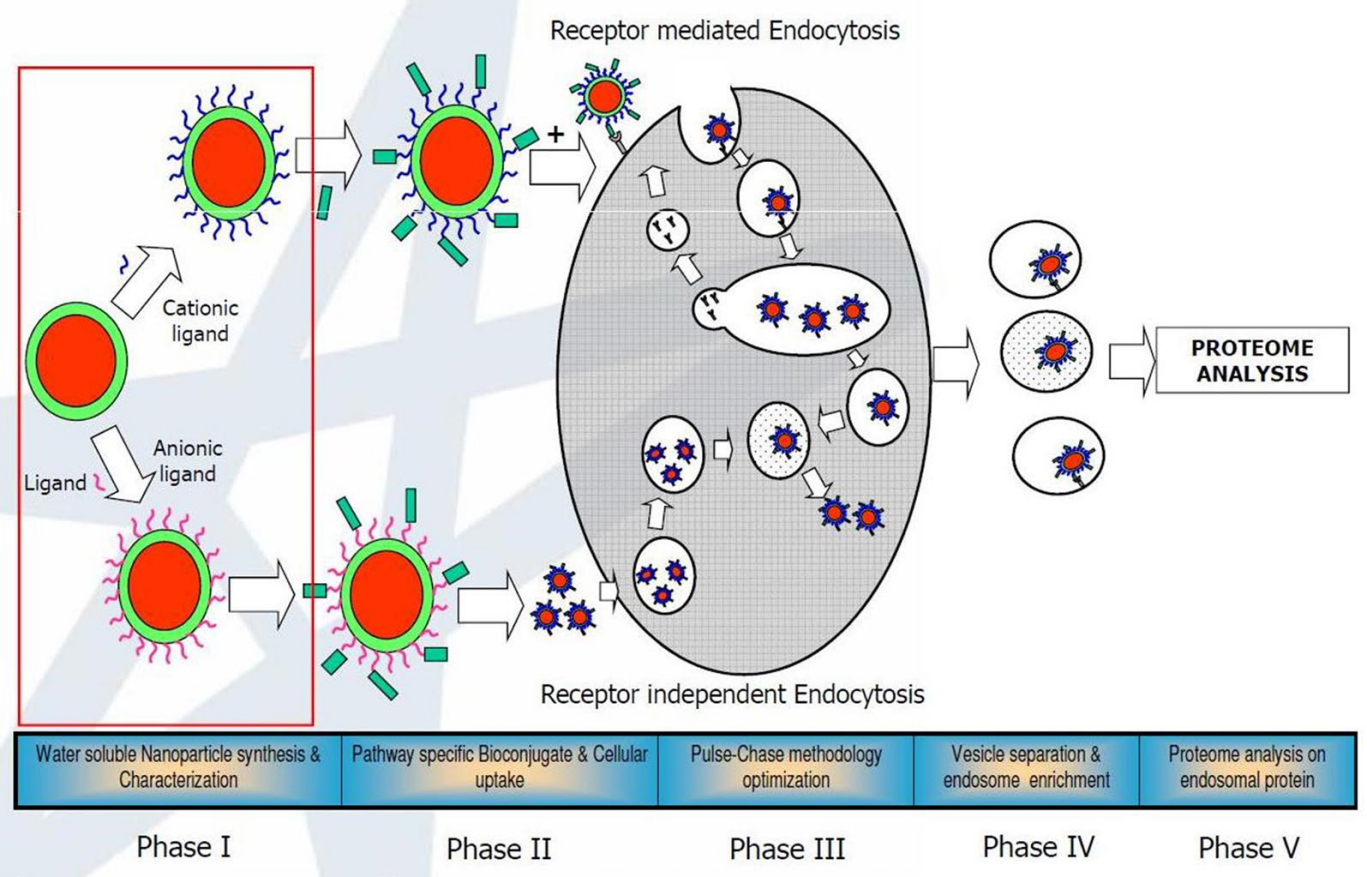
Step by step approach toward subcellular compartmental proteomics

**Fig. 7:**
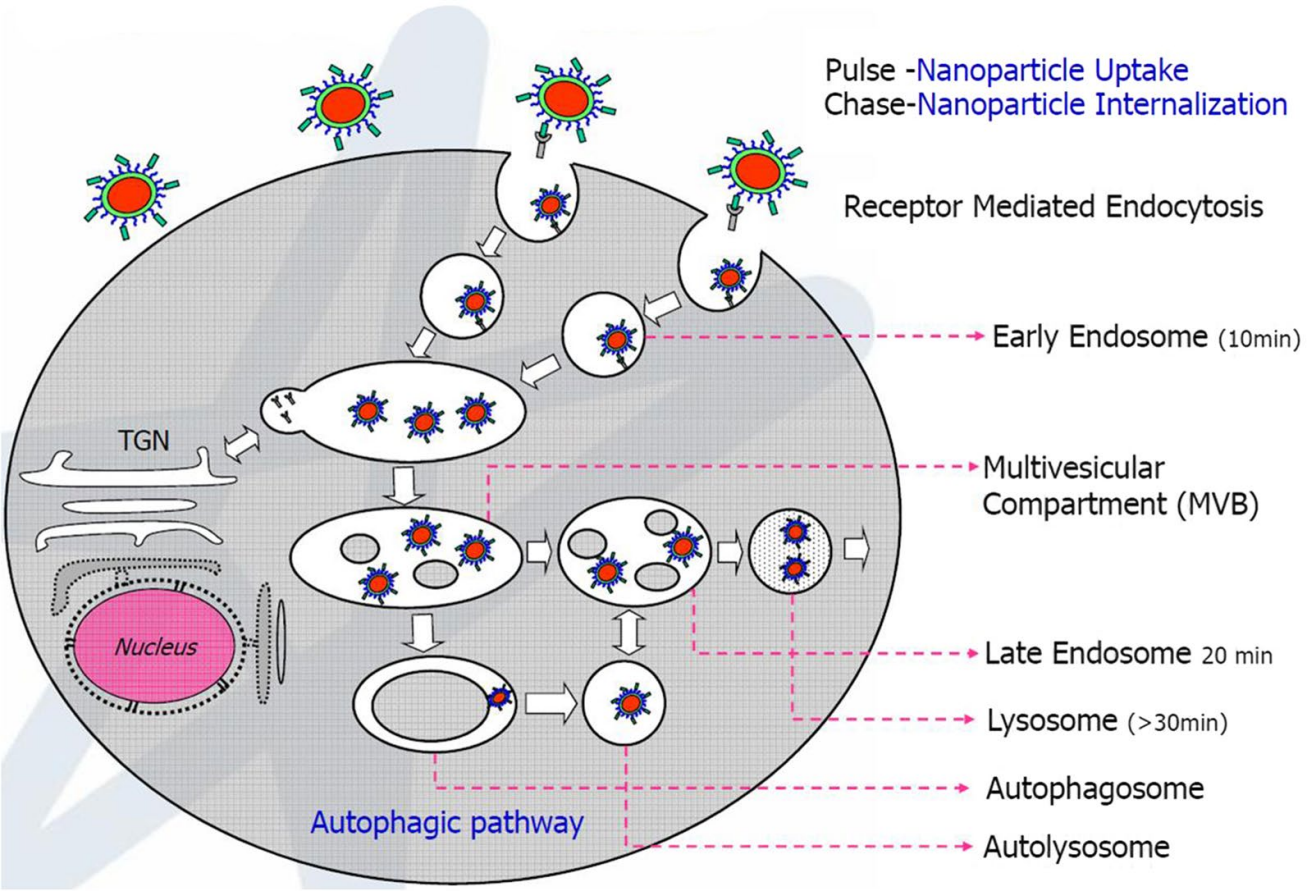
Pulse-chase methodology

Pulse-chase methodology is a commonly used approach to study the mechanism of endocytosis. Generally, pulse-chase strategy for omics analysis includes five stages or phases wherein *Phase-I:* includes generation of water-soluble nanoparticle by existing (thermal decomposition or chemical precipitation) synthesis and quality control using characterization; *Phase II:* includes selective bioconjugation of nanoparticle for a selective pathway-specific cellular uptake. For such pathway-specific cellular uptake, protein/ligand/synthetic peptide is used for receptor mediated endocytosis and charge dependent shell uptake is used for receptor independent endocytosis. *Phase III:* Pulse-Chase methodology is used to optimize pulse and chase period to selectively localize nanoparticle in vesicle. *Phase IV:* Magnetic separation strategy is used for subcellular compartmental enrichment along with ultracentrifugation. *Phase V:* Endosomal proteome using Mass Spectrometry analysis. This pulse-chase strategy was commonly used in radioactive labeling in the cell and this technique has now been extended to nanoparticle based subcellular compartmental isolation (Fig. 6). Briefly, pulse-chase strategy is used to govern receptor-mediated endocytosis of nanoparticle-ligand complex and has recently been extended to other endocytosis mechanisms [58]. During pulse period, nanoparticle is incubated with cells at 37 °C or at 4 °C for a certain period of time (0–1 h.) in the presence of medium. Depending on the application, nanoparticle with appropriate concentration is incubated in PBS or culture medium at 4 °C (for non-chase conditions) and 37 °C (for chase conditions). This time frame allows nanoparticle to interact with the cell surface and its protein. Depending on the dynamics and kinetics of nanoparticle-ligand interaction, pulse incubation involves a time period in the range of 10 min to 1 h. For example, if it is for cell membrane or plasma membrane isolation, the nanoparticle is incubated at 4 °C for 15–20 min in PBS with the adherent cells. Depending on whether the cells are adherent or in suspension, or it is receptor mediated or charge mediated, there is variation in the pulse time period required for cellular uptake [59]. After pulse period is performed, the chase is incubated at appropriate time period depending on the compartmental isolation. Chase period represents the time where the nanoparticle containing medium is replaced with fresh medium without nanoparticle. This supports streamlining nanoparticle internalization in the cell and accumulation of nanoparticle into a certain compartment of interest depending on the timeframe. For endosomal isolation, chase period is generally for a timeframe of 10–15 min. For late endosomes, chase period is generally for 15–20 min and for lysosomes it is more than 30 min. However since endocytosis is dynamic in mechanism, it is relatively difficult to isolate highly pure early and late endosomes (Fig. 7). At the same time it is possible to isolate highly pure lysosome by performing a chase period of more than 3 h and up to 24 h. This is mainly because lysosome is the endpoint for most of the endocytosis [60]. For targeting, phagosome or autophagosome, chase period is adjusted accordingly for 30–60 min before phagosome fuses to lysosome. Nanoparticle can be concentrated in lysosome after 60 min of chase period. An advantage of using chase period is that it provides useful information for nanoparticle tracking. For this reason, fluorescence tagged nanoparticle is used for pulse–chase methodology and live-cell imaging [61]. By incubating with endocytic inhibitors for endosomal or lysosomal fusion, it is possible to limit the nanoparticle-cellular internalization and subcellular trafficking. For example, by limiting the endosome-lysosomal fusion using Latrunculin-A, it is possible to concentrate the nanoparticle in early or late endosomes [62]. It is also reported that the nanoparticle coupled ligand does not mimic the ligand cellular uptake and subcellular compartment localization. Few examples in the later part of this article elaborate on the deviation in cellular uptake. These examples are confirmed by using pulse-chase methodology, magnetic organelle isolation and live cell imaging using fluorescence tag [63]. For optimal use of pulse-chase method, it is important to establish a methodology for specific subcellular compartments. Here, we describe two interesting nanoparticle based methodologies to isolate plasma membrane and endosomal compartments using affinity purification.

## Nanoparticle based novel method for plasma membrane affinity purification

**Fig. 8:**
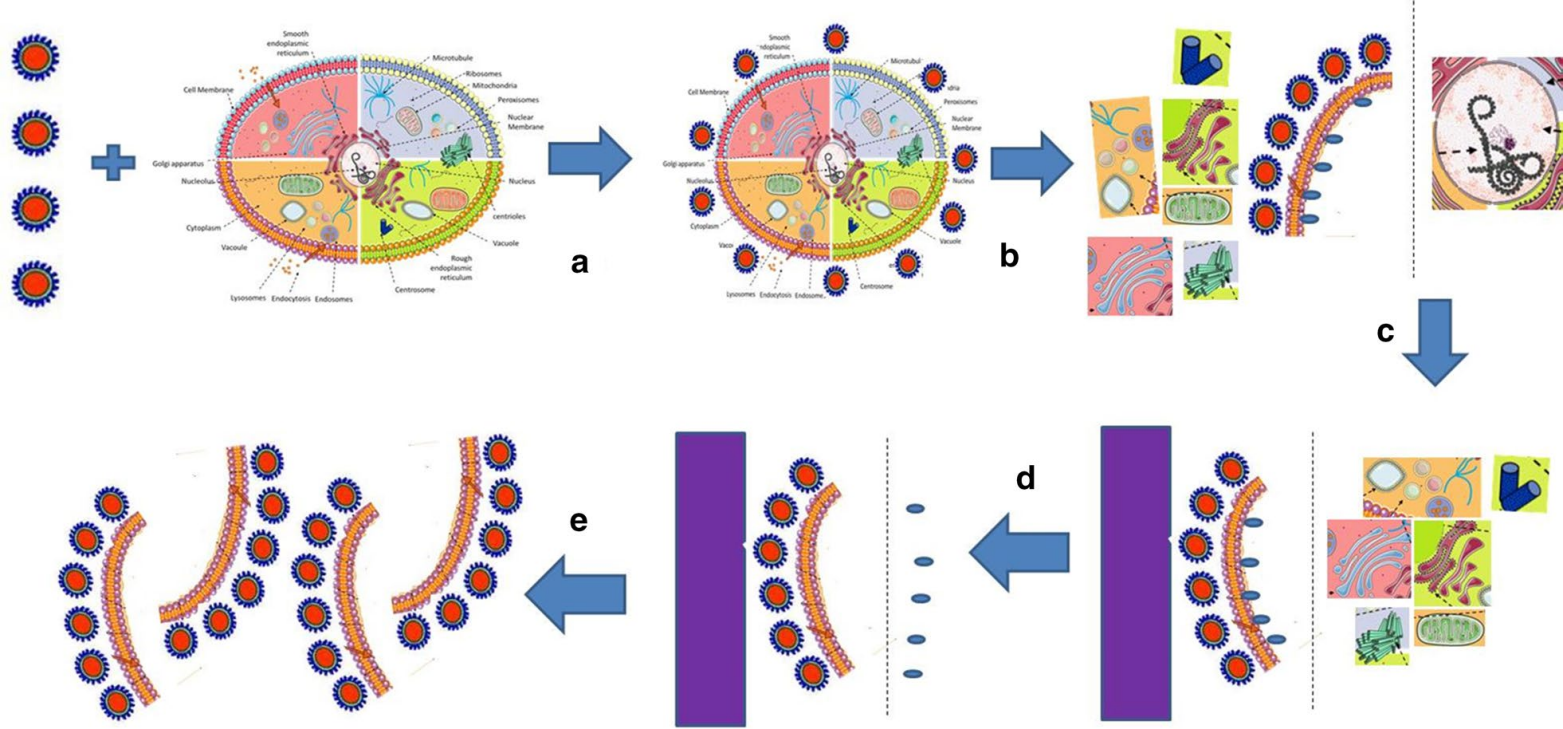
Strategy towards Plasma Membrane isolation

Figure 8 represents the step-by-step strategy to isolate plasma membrane using nanoparticle based affinity purification. Briefly, SPMNPs coated with positively charged NH_2_ functionalized PEGylated lipids are incubated with adherent cells grown in a 10 cm dish or a 75 cm^2^ flasks. The time period of incubation is generally 15 to 20 min at 4 °C in PBS with horizontal shaking such that it doesn’t detach the cells. (Note: An additional step of incubating adherent cells at 4 °C for 30 min before nanoparticle-cell interaction is recommended). After incubation, the nanoparticle containing supernatant is removed and washed twice with fresh ice-cold PBS. Cells are removed from the dish/flask by scrapping the cells. Detachment of cells using trypsin is not recommended as it might affect cell surface proteins which this method aims to isolate. Cell suspension is further homogenized using a homogenizing apparatus and buffers that maintain physiological conditions. Further, nuclear fraction and unbroken cells are separated from post nuclear fraction by centrifuging at 800 rpm for 10 min at 4 °C. The post nuclear fraction is passed through the magnetic field. Here, the unbound fraction is eluted while the magnetic fraction is further washed with 1 M potassium chloride and 0.1 M sodium carbonate solution in the presence of magnetic field. An additional washing step with homogenizing buffer can be included to further clear the unbound material. 1 M KCl and 0.1 M Na_2_CO_3_ solution are used to remove cytoskeleton-associated compartments from the cell surface proteins. Further, the magnetic field is removed and bound fraction is eluted from the column. Finally the bound fraction is enriched by pelleting at 50,000 rpm for 1 h. The pellet is resuspended in an appropriate amount of PBS for further analysis like mass spectrometry.

## Nanoparticle based novel method for endosomes and lysosomal affinity purification

**Fig. 9:**
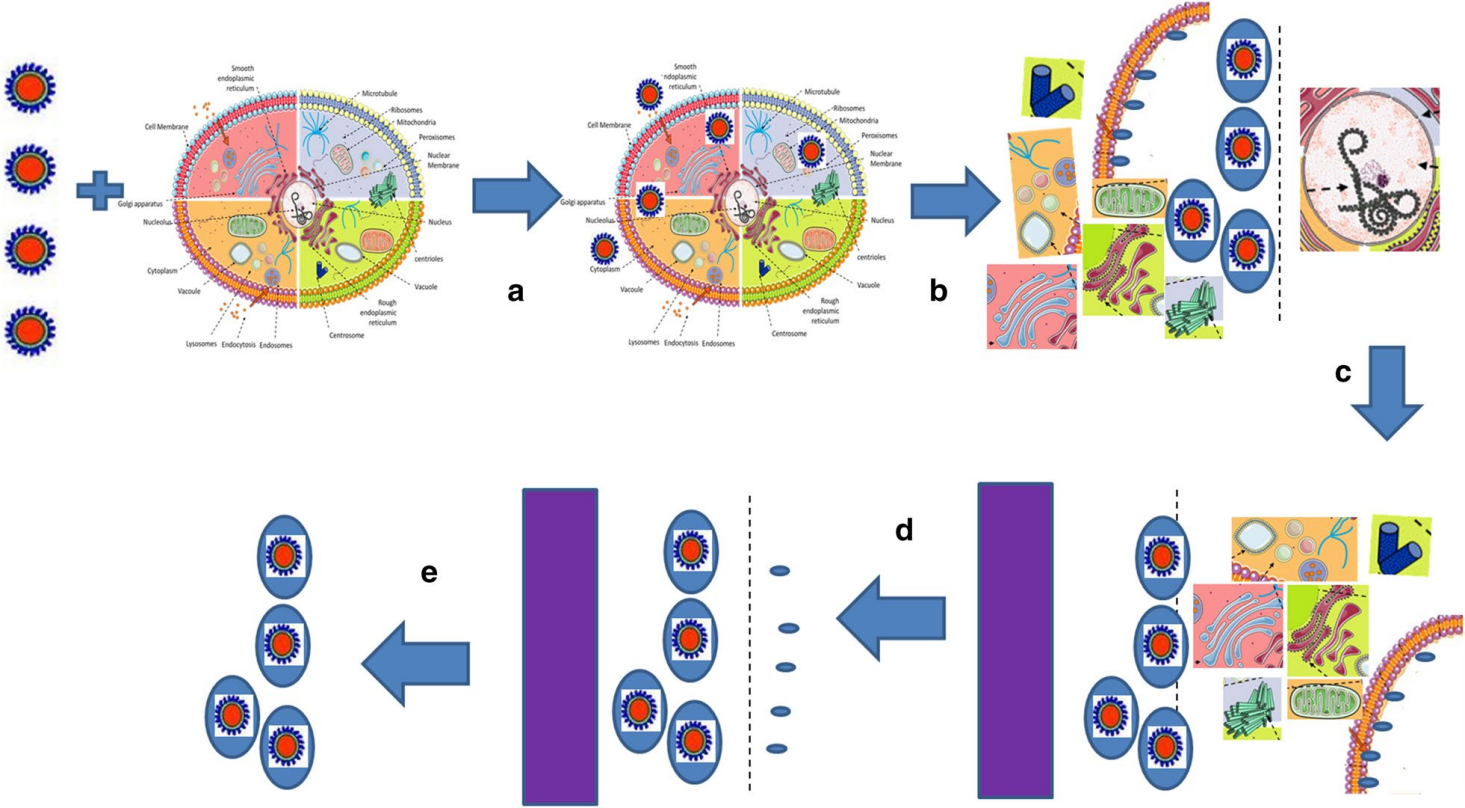
Strategy towards endosomal isolation

Figure 9 represents another step-by-step strategy to isolate endosomes and lysosomes using nanoparticle based affinity purification. Briefly, DMSA or TMAOH or Silane coated nanoparticles are incubated with adherent cells that are grown in 10 cm dishes. The pulse time period is generally 15 to 20 min at 37 °C in medium with horizontal shaking such that it doesn’t detach the cells. After incubation, the SPMNPs containing medium is removed, washed twice with fresh medium and incubated for the chase period (0–24 h) with fresh medium. Cells are then removed from the dish/flask by scraping. Cell suspension is further homogenized as explained in the previous section. Further, nuclear fraction and unbroken cells are pelleted at 800 rpm for 10 min at 4 °C. The post nuclear fraction is passed on the column in presence of magnetic field. Here, unbound fraction is eluted while the magnetic fraction is further washed with the homogenizing buffer to further clear unbound material. This is followed by the removal of magnetic field and elution of the bound fraction from the column. Finally the bound fraction is enriched by pelleting at 50,000 rpm for 1 h. The pellet is resuspended in PBS for further analysis. Based on chase, the subcellular localization of nanoparticles can be determined as illustrated Early (~10 min), Late (~20 min) and Lysosomes (>30 min) [57, 58, 64–68].

Although nanoparticle-protein complex can be used for endosomal trafficking and for proteomics, there are illustrations, which show that nanoparticle-protein complexes are trafficked differently compared to the target protein complex. For example, trafficking of ricin conjugated nanoparticle is reported to be unlike the ricin ligand where trafficking occurs from early endosomes (EE), trans-Golgi network (TGN) and finally to endoplasmic reticulum (ER). It is well known that transferrin is recycled to the cell surface via recycling endosomal compartments such as recycling endosome via early endosomes and multi-vesicular bodies. However, transferrin conjugated nanoparticle and Shiga toxin (ricin) conjugated nanoparticle are shown to traffic from early endosomes to late endosomes and finally accumulated at lysosomes. It is also reported the Shiga toxin conjugated nanoparticle tend to accumulate at early endosomes while Shiga toxin traffic like ricin (from EE to TGN and finally to ER).

## Conclusion

Tables 2 and 3 list the commonly used techniques for subcellular isolation of plasma membrane and lysosomes with high purity and yield. Both tables show that nanoparticle based methods hold many advantages as compared to existing methods. Using the isolation technology, several omics datasets for subcellular compartments can be generated for any given cell. There is an interesting aspect in the use of nanoparticle based method that is generic in nature. Hence, the method can be applied to wild-type and diseased cell-type (for example cancer cell) for plasma membrane and endosomal compartmental isolation. By using the nanoparticle based subcellular compartmental isolation; one could potentially generate a complete and comprehensive plasma membrane or endosome or lysosome proteomics, glycomics and lipidomics for any cell type. By the generated subcellular omics, nanobiotechnology can serve as a useful tool to build omics datasets for cancer biology using the bottom-up pyramid approach. Using the bottom-up pyramid approach in omics analysis, subcellular omics datasets (including genomics, proteomics, lipidomics and glycomics) can be compiled together and compared with the whole cell omics analysis. This approach can also be used to generate several omics datasets in cancer biology that can enable the researchers to revisit the subcellular omics in order to understand the biological significance and functional relevance. For all such omics analysis studies, it is necessary to have an efficient, robust and high precision technology for subcellular compartmental isolation. The technology also needs optimization and fine tuning depending on its applicability with host cell system. Using different nanobiotechnology tools for subcellular compartmental isolation, several high-throughput functional omics dataset like fluxomics, metabolomics, interactomics and localizomics can be generated. Using all these datasets, comprehensive Phenome and subcellular omics are generated that can by analyzed using nanotechnology for data storage studies. Further dataset thus gets larger for different diseases such as cancer, diabetes, infectious diseases, ageing related diseases, and neurodegenerative diseases. These dataset and nanotechnology based analytics can be used in drug development, pre-clinical studies, patent analytics, and other applications. As a future perspective, the use of nanoparticle as nanobiotechnology tool is all set to be a game changer in the generation of Datasets for systems biology.
